# Better vertebrae remodeling in pediatric spinal eosinophilic granuloma patients treated with kyphoplasty and short-term posterior instrumentation: A minimal two-year follow-up with historical controls

**DOI:** 10.3389/fped.2022.922844

**Published:** 2022-11-23

**Authors:** Yiming Zheng, Zhiqiang Zhang, Dahui Wang

**Affiliations:** Department of Pediatric Orthopaedics, Children's Hospital of Fudan University, National Children's Medical Center, Shanghai, China

**Keywords:** kyphoplasty, eosinophilic granuloma, pathological vertebral body fracture, vertebral remodeling, child

## Abstract

**Objective:**

To assess the validity and safety of kyphoplasty combined with short-term posterior instrumentation to treat children with vertebrae plana due to eosinophilic granuloma (EG).

**Patients and Methods:**

Clinical data of EG patients, who received kyphoplasty and short-term instrumentation from March 2019 to March 2020, were retrospectively reviewed. The recovery of diseased vertebrae was assessed and compared with historical case data.

**Results:**

Nine patients with EG had received kyphoplasty and short-term posterior instrumentation. The mean age at initial treatment was 66.7 months old (range, 28–132 months). The average number of follow-up months was 26.7. (range, 24–30 months).Four and 5 cases presented with thoracic and lumbar vertebral destruction, respectively. Under Garg's classification, 7 and 2 cases were classified as Grade IIA and IIB, respectively. The average diseased vertebral heights at 1-year and 2-year after surgery were significantly higher than the preoperative heights. The average percentages of diseased vertebral heights to references at 1-year and 2-year after surgery were 72.0% and 86.0%, respectively. The average percentage of diseased vertebral heights to the references at 2-year after surgery was significantly higher than that of the historical cases at the same time. No minor or major adverse events were observed.

**Conclusions:**

Transpedicular balloon kyphoplasty for the direct restoration of vertebrae plana seems feasible and safe in combination with short-term posterior instrumentation. Better short-time vertebrae remodeling was observed 2 years after surgery. Active surgical treatment is suggested for children who have vertebrae plana as a result of EG in order to maintain the ability to recover vertebral height.

## Introduction

Eosinophilic granuloma (EG), distinguished by a clonal expansion of Langerhans-type cells with bone involvement, is the most predominant condition in the clinical spectrum of Langerhans-cell histiocytosis (LCH) (1, 2). Children or young adults tend to experience EG more frequently (1–4). It can affect any bone in the skeleton. EG makes up 6.5%–25% of all spinal bone tumors in the spine (4–8). In young children, EG is frequently the cause of vertebral damage (9). An osteolytic region that causes vertebral collapse or incomplete collapse, also known as vertebra plana, makes up the characteristic vertebral lesion of EG. A pathological fracture or spinal instability with significant mechanical back pain may result from the bone destruction of vertebral EG. EG treatment choices differ according to the severity of the disease (1). Surgery should be considered for patients who have a spinal deformity, neurological impairments, or a poor response to conservative treatment (10, 11). Several surgical strategies have been documented to be feasible, but no consensus has been reached.

In our institution, patients with EG, whether or not the spine was involved, were primarily treated with chemotherapy following percutaneous biopsy. For patients who have a spinal deformity or neural deficits, biopsy, focal curettage and temporary pedicle screw fixation was the most common surgical strategy and acquired good results. But for the severe cases, the restoration of vertebral height did not come up to expectation (12).

Balloon kyphoplasty (BKP) is an image-guided surgery in which the damaged vertebra is first inflated with a balloon to treat kyphosis before being injected with cement to stabilize the spinal column (13). BKP is often used to treat osteoporotic vertebral compression fractures and has achieved significant results (14–18). And studies (19, 20) on pathological fractures have reported that the BKP technique had a superior functional outcome and few adverse events. Nine patients with vertebral plana because of spinal EG have had effective BKP combined with short-term posterior instrumentation at our facility.

The purpose of this early report was to demonstrate the validity and safety of BKP in combination with short-term posterior instrumentation in a small-scale investigation of nine patients with vertebrae plana due to spinal EG.

## Patients and methods

### Participants

The author's institution's ethical committee authorized this study plan, and it was carried out following the guidelines in the Declaration of Helsinki for medical research involving human participants.

We retrospectively reviewed all patients with spinal lesions between March 2019 and March 2020 in our institution. Inclusion criteria included (1) histological examination confirmed vertebral EG; (2) treated with BKP in combination with short-term pedicle screw instrumentation. Exclusion criteria included (1) presence of congenital spinal malformations and (2) spine surgery history. The FLACC and Frankel scales were used to assess pain and neurological function, respectively. The patients received routine skeletal plain radiography examinations, local computed tomography (CT), magnetic resonance imaging (MRI), and whole-body bone scintigraphy preoperatively. At each return visit, spine plain radiography was performed.

For a reference to typical vertebral height, the vertebrae next to the upper instrumented vertebrae were chosen (to eliminate the effect of internal fixation). By averaging the anterior and posterior heights of the vertebral body, the height of the diseased and reference vertebrae was determined.

Based on our clinic database, we established a historical control cohort for comparison, taking into account all spinal EG patients who fit both the inclusion and exclusion criteria listed above but who were only given temporary short-segment posterior instrumentation prior to chemotherapy.

The radiographic classification of the maximal vertebral collapse was done using Garg's Classification System (21). Grade-I lesions collapse between 0% and 50%, while grade-II lesions collapse between 51% and 100%. Based on the morphology of the vertebral collapse, the lesion is also categorized as A (symmetric) or B (asymmetric). Lesions of grade III do not fall into the A or B subcategories and affect the posterior components of the spine.

### General management of EG

In our hospital, patients with bone lesions will receive a percutaneous or/and an open biopsy after the preliminary evaluation. Once the histological diagnosis is confirmed as EG, immobilization and chemotherapy are the first-choice treatments. The patient will be referred to hematologists to get chemotherapy.

Patients with (1) vertebral body compression greater than 50% and (2) spinal instability [evaluated by the Spinal Instability Neoplastic Score, SINS ≥7 (22)] after conservative treatment for 3 months but the symptom progress, or (3) neurological symptoms will receive surgery.

### Operative procedure

**Figure 1:**
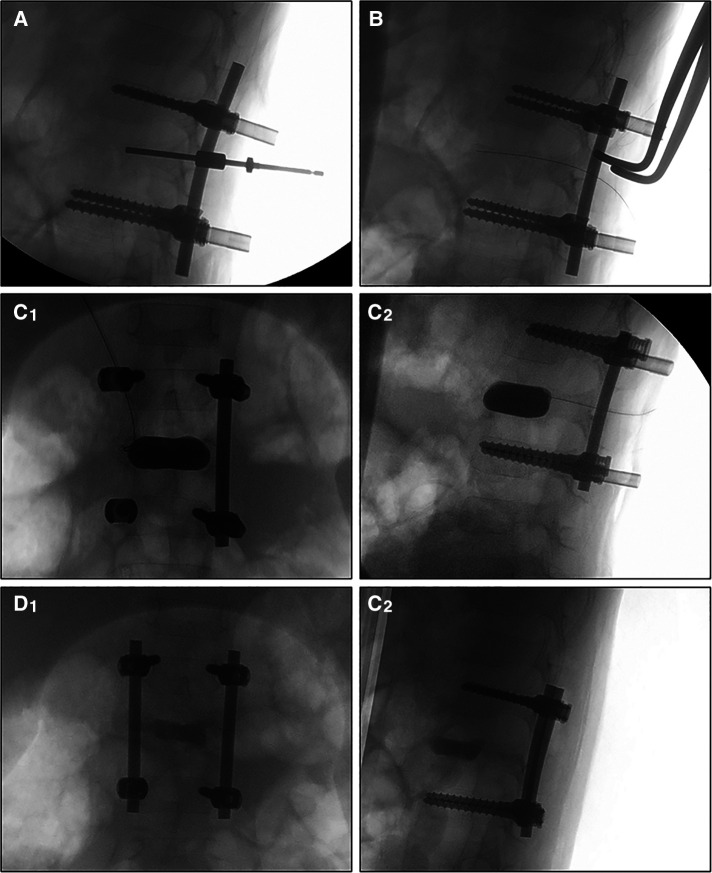
Fluoroscopy images during the operation. The work tunnel was created transpedicular (**A**), then the balloon was loaded into the vertebral body (**B**) after the lesion curettage and inflated simultaneously under fluoroscopic guidance and pressure control (up to 120 psi) (**C1,C2**). The void was filled with calcium sulfate cement and then the rods were locked (**D1,D2**).

Patients were positioned in a prone posture while under general anesthesia. A transpedicular percutaneous biopsy was performed. The lesions were sent to the Department of Pathology for rapid pathological examination. The operation was suspended, usually for 15-minute duration, until the diagnosis was confirmed. Then, the pedicles of the diseased and adjacent vertebrae were exposed through a midline incision, avoiding injury to the facet joints and local ligaments. Four monoaxial pedicle screws were implanted in the adjacent vertebrae. and temporarily locked up with physiological arc pre-bent rods. After that, a tunnel was created through the pedicle of the diseased vertebra. Through the tunnel, the lesion curettage and neurological decompression were carried out. The vertebral epiphyses were maintained and protected with great care. Then, after loosening the screw caps, an access cannula was placed in the transpedicular tunnel to create a channel for balloon placement. An 8- or 12-mm-diameter balloon, according to the size of the vertebral body, was placed into the vertebral body through the channel and inflated simultaneously under fluoroscopic guidance and pressure control (up to 120 psi). Usually, as there was already a cavity created by lesion curettage, it was easy to place the balloon into the center of the vertebra body by unilateral transpedicular approach. During balloon inflation, special care should be taken not to fracture the end plates or lateral walls. Once the vertebra kyphosis has been corrected, the balloon was deflated and removed after 3-minutes of maintenance. Then the screw caps were locked up again, and the balloon was deflated and removed. The void was filled with calcium sulfate cement (CSC) injected at low pressure, under fluoroscopy guidance (Figure 1). No fusion was performed.

If the rapid histological diagnosis was negative or uncertain, a second-time biopsy and rapid pathological examination would be performed. If the result was still negative or uncertain, a third-time biopsy would be performed and the sample would be sent for regular histological examination. The operation would be ended. Further treatment depends on the regular histological diagnosis, which is usually due in 5–7 days.

Patients of the historical control cohort received the same operation but without the balloon kyphoplasty and CSC injection.

Through the initial incision, internal fixation removal surgery was performed on patients who had completed chemotherapy and had been monitored for more than a year with vertebral height recovery of more than 50% to the reference.

### Postoperative care and follow-up

Patients were recommended to start daily activity out as soon as feasible after the initial surgery. A brace was used for 3 months. According to the DAL-HX90 regimen (23), a hematologist started chemotherapy two weeks following surgery. Follow-up visits to the orthopedic clinic were scheduled every three months in the first year and, after that, every 6 months.

### Statistical analysis

To make data easier to grasp, continuous data in descriptive statistics are presented as the mean ± standard deviation, and categorical data are expressed as frequencies. The paired *t*-test was applied to compare the quantitative variant distributions intra-cohorts. The Independent *t*-test was applied to compare the quantitative variant distributions inter-cohorts. When *p* < 0.05, significance was considered. SPSS 22.0 software was used for statistical analysis.

## Results

### Common clinical characteristics of treated patients

Nine patients (6 boys and 3 girls) with EG had received kyphoplasty and short-term pedicle screw fixation (Table 1). The mean age at initial treatment was 66.7 months old, ranging from 28 to 132. Five occurrences of lumbar vertebral damage and four cases of thoracic vertebral destruction were present. 7 and 2 cases, respectively, were assigned the grades IIA and IIB in Garg's classification. All of the patients had mild to moderate back discomfort before the procedure, which was aggravated at night. Six cases had weakness of the lower limbs.

**Table 1:** Patients’ characteristics of BKP cohort.

No.	Sex	Age (months)	Diseased vertebrae	Grades	FLACC	SINS	Frankel	WBB zones
1	M	36	L3	IIA	4	11	D	4–9
2	M	96	T12	IIA	5	12	D	4–9
3	M	34	L4	IIB	3	10	E	4–9
4	M	132	T8	IIA	6	10	D	4–9
5	F	98	L2	IIA	4	11	D	4–9
6	M	61	T6	IIB	4	9	D	4–9
7	F	76	T7	IIA	4	11	D	4–9
8	M	28	L4	IIA	3	9	E	4–9
9	F	39	L4	IIA	4	9	E	4–9

**Figure 2:**
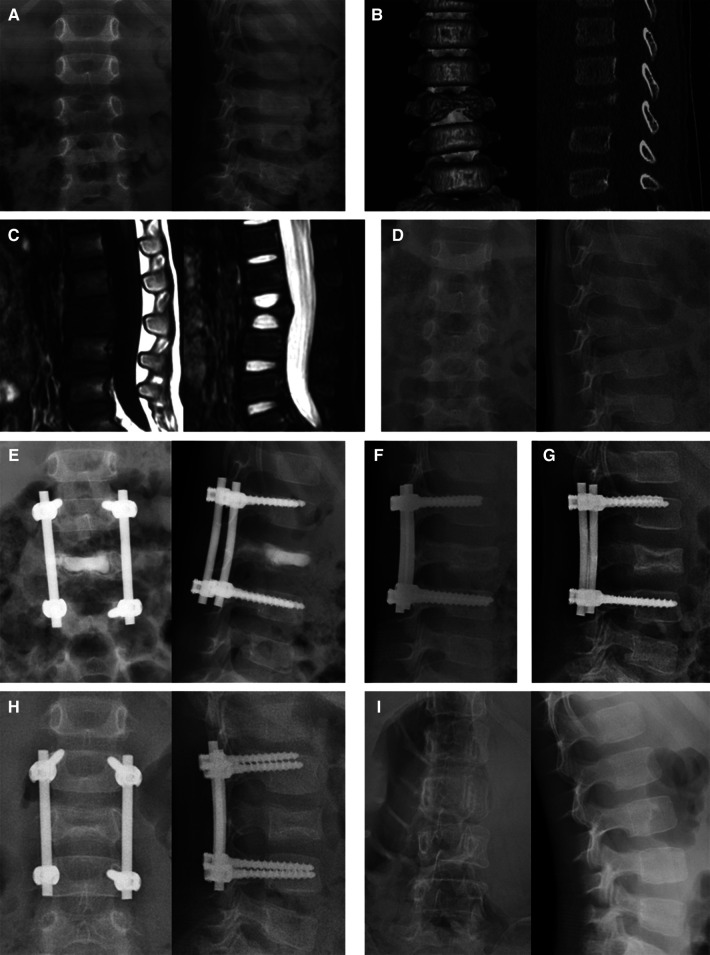
Series images of case 1, a 36-month-old boy with L3 vertebra plana. The patient took the plain x-ray (**A**), CT (**B**), and MRI (**C**) in the local hospital 2, 3, and 4 weeks after the symptom of back pain presented, respectively. These series images showed the aggravating damage of the L3 vertebra body. Two months after the initial symptom, the x-ray image (**D**) showed Grade IIA destruction of the L3 vertebral body. Immediately after the operation, vertebral height was partly restored (**E**). The calcium sulfate cement was completely absorbed in the 3-month follow-up (**F**). The vertebral height increased at the 6-month follow-up and osteosclerosis was observed (**G**). One year after the operation (**H**), an 89.2% recovery of vertebral height was observed and then the internal fixations were removed. Two years after the first operation (**I**), the vertebral body was well remodeled.

Within the first year, follow-ups were done every three months, and then every six months after that. The average follow-up time was 26.7 months, varying from 24 to 30 months. At each follow-up appointment, plain radiography exams were conducted and the heights of sick and referred vertebrae were measured (Figure 2).

### Evaluation of the surgical strategy

**Figure 3:**
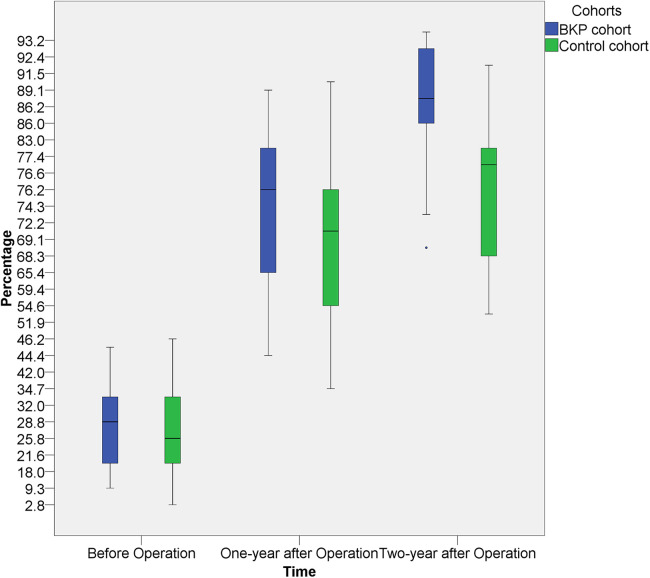
The heights of the diseased vertebral at 1-year and 2-year after surgery showed a significant increment, both intra the BKP cohort and the control cohort. The average recovery speed was higher in the first year. No significant inter-cohort height difference was observed at 1-year post-operation, but better vertebral height restoration was observed in the BKP cohort at 2-year post-operation.

The average operative time was 134 ± 21 min, the average blood loss was 53 ± 24 ml (Table 2). All of the patients' back pain symptoms disappeared within two weeks of surgery, and the six cases of lower limb weakness subsided between two and four weeks after surgery. The vertebrae heights were restored immediately after the operation. In general, the diseased vertebrae displayed a trend in the recovery of vertebral heights after surgery, even though recovery rates varied by patient and vertebra. The paired t-test on the average of preoperative diseased vertebral heights and vertebral heights at both 1-year and 2-year after surgery showed a significant increment (*p* < 0.001). The recovery speed was higher in the first year (Figure 3).

**Table 2:** Surgical details and follow-up features of BKP cohort.

No.	Operative time (minutes)	Blood Loss (mL)	VH0 (%)	VHp (%)	VH1 (%)	VH2 (%)	Complications	Last time Follow-up (months)
1	112	80	20.5	86.2	89.1	96.0	None	30
2	142	50	32.0	91.1	81.3	93.2	None	30
3	148	30	21.6	86.5	76.2	86.9	None	30
4	133	50	33.3	53.4	74.3	86.0	None	30
5	170	100	45.2	83.0	86.2	92.5	None	24
6	148	50	28.8	58.3	76.6	86.1	None	24
7	132	60	9.3	59.0	54.6	72.8	None	24
8	98	20	43.4	87.1	65.4	92.4	None	24
9	124	40	18.0	62.9	44.4	68.5	None	24

VH0: percentage of diseased vertebral body height to reference, before operation.

VHp: percentage of diseased vertebral body height to reference, immediately after operation.

VH1: percentage of diseased vertebral body height to reference, 1-year after operation.

VH2: percentage of diseased vertebral body height to reference, 2-year after operation.

With the historical cohort, 11 patients (8 boys and 3 girls) were included (Table 3). There was no significant difference in age, sex, grades, WBB zones, and operation time between the two cohorts. In comparison with the historical cohort (Table 4 and Figure 3), no significant difference was observed at 1-year post-operation (*p* = 0.387), but better vertebral height restoration was observed in the BKP cohort (*p* = 0.027) at 2-year post-operation. By the last follow-up, no significant complications or additional surgeries were recorded, and no recurrence was recorded.

**Table 3:** Clinical details of the historical cohort.

No.	Sex	Age (months)	Diseased vertebrae	Grades	SINS	Frankel	WBB zones	Operative time (minutes)	VH0 (%)	VH1 (%)	VH2 (%)	Complications	Last time Follow-up (months)
1	M	56	L5	IIA	11	D	4–9	180	21.6	51.8	59.4	None	72
2	M	56	T10	IIA	9	D	4–9	120	2.8	34.7	53.9	None	60
3	F	142	L3	IIA	10	E	4–9	99	30.0	72.2	77.4	None	60
4	M	25	T11	IIB	11	D	4–9	120	10.6	89.2	91.8	None	60
5	F	118	L4	IIA	12	D	4–9	141	28.6	51.9	68.3	None	59
6	M	78	T7	IIA	11	D	4–9	124	22.8	56.3	69.1	None	60
7	M	31	L3	IIA	12	D	4–9	102	42.0	83.3	91.5	None	48
8	M	130	T5	IIA	9	E	4–9	70	8.6	62.6	67.0	None	50
9	F	119	T4	IIA	9	E	4–9	186	41.1	70.5	76.9	None	48
10	M	18	T10	IIA	9	E	4–9	98	25.8	75.4	77.4	None	36
11	M	97	T9	IIA	9	E	4–9	94	46.2	76.4	83.0	None	36

VH0: percentage of diseased vertebral body height to reference, before operation.

VH1: percentage of diseased vertebral body height to reference, 1-year after operation.

VH2: percentage of diseased vertebral body height to reference, 2-year after operation.

**Table 4:** Clinical data of BKP cohort in comparison with the historical control cohort.

		BKP cohort	Historical cohort	*p*-Value^a^
Number		9	11	
Age (months)	Mean	66.7 ± 36.2	79.1 ± 44.7	*p* = 0.511
	Range	28–132	18–142	
Sex	F	3	3	*p* = 1.000
	M	6	8	
Garg's classification	IIA	7	10	*p* = 0.566
	IIB	2	1	
Operation duration (minutes)	Mean	134 ± 21	121 ± 36	*p* = 0.357
	Range	112–170	70–186	
VH0 (%)	Mean	28.0 ± 11.9	25.5 ± 14.2	*p* = 0.673
	Range	9.3–45.2	2.8–46.2	
VH1 (%)	Mean	72.0 ± 14.7	65.8 ± 16.1	*p* = 0.387
	Range	44.4–89.1	34.7–89.2	
VH2 (%)	Mean	86.0 ± 9.5	74.2 ± 12.1	*p* = 0.027
	Range	68.5–96.0	53.9–91.8	

VH0: percentage of diseased vertebral body height to reference, before operation.

VH1: percentage of diseased vertebral body height to reference, 1-year after operation.

VH2: percentage of diseased vertebral body height to reference, 2-year after operation.

^a^ Independent t-test for comparison of quantitative variant distributions inter-cohorts; Fisher's exact test is used to compare categorical distributions, while paired t-tests are used to compare quantitative variation intra-cohort distributions.

## Discussion

The treatments of spinal EG remain controversial. The use of observation and immobilization, acesodyne medicine, chemotherapy, radiation, and local excision or curettage with or without bone grafting are just a few of the described therapeutic options (1, 24, 25). First of all, we recommend undergoing a biopsy for histological diagnosis and disagree with the so-called “wait-and-see” strategy. Although EG is frequently the cause of vertebral plana in children, other factors must be taken into consideration (9). Patients, especially children with symptomatic bone lesions should not be left alone to let the disease take its natural course without a histological diagnosis. Then, in general, the treatment of typical solitary lesions in asymptomatic patients is conservative, namely, observation and immobilization (26, 27). Vertebral destruction is a frequent EG symptom, however, the endplates of the vertebrae are typically untouched by the disease process. As a result, the vertebral body will continue to develop with time and have the ability to rebuild itself (22, 28, 29). Younger children have the best chance of successfully undergoing vertebral body restoration (28).

Currently, the regimen of vinblastine and prednisone is a first-line treatment for multisystem LCH (1). And chemotherapy in single-site EG has also been reported (30, 31). Especially vertebral lesions were managed by chemotherapy to prevent scoliosis or spinal deformity (32). In our hospital, patients with EG, whether or not the spine was involved, were primarily treated with chemotherapy following percutaneous biopsy. No radiotherapy was used in our cohorts, although some authors have highlighted radiotherapy in spinal EGs (33, 34). Ionizing radiation can harm bone development in children and increases the risk of secondary cancer, radiation myelitis, and damage to the vertebral enchondral plate, all of which can harm growth.

Although solitary eosinophilic granuloma is regarded as a benign lesion, for patients with spinal deformity, and/or persistent spine instability and neurological symptoms, surgical treatment should be adopted (10, 11, 35–38). Surgery is used to confirm the diagnosis, preserve the vertebral body's normal anatomical structure and function, and treat neurological symptoms. Hence, for the patients who met the aforementioned surgical indications, surgery treatment was adopted. Some researchers recommend that these individuals be treated with radical or margin excision surgery, followed by bone grafting, fusion, and internal fixation (10, 39, 40). However, with a growing child, the normal development of the fused vertebra will be limited by the premature fusion of the spine. As a result, interbody fusion was not employed in our procedure.

Kyphoplasty, the variation of the standard percutaneous vertebroplasty (PVP), was introduced to everyday clinical practice to stabilize the vertebral fracture, recover the height of the spine, and correct the associated kyphosis by Garfin, Reilley, and Lieberman (13, 41). A detailed description of both techniques can be found in CIRSE guidelines on percutaneous vertebral augmentation (42). Both PVP and BKP have been widely used for vertebrae augmentation with minimal surgical invasion, and their beneficial effects were stable and similar (43). However, a thorough investigation of their impact on pediatric vertebral plana caused by EG is lacking. In this study, nine EG patients have undergone BKP. the vertebral height and kyphosis had immediately restored and an average of 72.0% and 86.0% recovery at 1-year and 2-year follow-up, respectively. The back pain and neurologic impairment were relieved in 2-weeks and 2–4 weeks after surgery, respectively. The most frequent BKP complications include cement leakage, infection, pedicle or rib fracture, hemorrhage, allergic reaction, and neighboring vertebral body collapse (42). In our study, no mild or significant adverse events relating to the procedure were noticed during the observation period, which might be related to the unbroke endplate, the rich surrounding ligaments, and the low inflation pressure.

Poly-methyl-methacrylate (PMMA) is the most common cement used in BKP. It is affordable, simple to work with, permits the combination of radiopaque materials, and provides the vertebral body with the proper rigidity and strength. However, because it lacks osteoinductive or osteoconductive qualities, it won't eventually osseointegrate with the host bone (44). As potential candidates for PMMA substitutes, calcium sulfate was used as a regenerative material as early as the late 19th century (45). It is well-tolerated when used to fill bone defects (46, 47) and undergoes rapid and complete resorption without eliciting a significant inflammatory response (48, 49), even if been grafted into the epiphysis and metaphysis zone (49). According to Lillo and Peltier, the calcium sulfate completely vanished in 45–72 days, and the bone abnormalities completely healed in around 3 months (45, 50). The CSC was also proved to be an effective bone substitute used in spinal surgeries (51). In individuals with osteoporotic vertebral compression fractures, it can successfully reduce pain and regain spinal strength as PMMA (52–54). Thus, in this study, we used the CSC to fill the vertebral void. With all 9 patients, the CSC was absorbed completely at the first follow-up and no adverse events were observed.

Due to its retrospective design and the fact that we only looked at nine instances from one institution with brief observations, this study has its limitations. The patient's selection bias may cause statistical errors. Furthermore, the height restoration varied among lumbar and thoracic cases, for which we can't carry out the reason analysis because small case number. More instances need to be gathered, and they need to be examined long-term and prospectively.

## Conclusions

The findings imply that surgical intervention is advantageous for EG patients with symptoms like a spinal deformity or neurological symptoms who failed conservative treatment. Taking a biopsy and treating the lesion right away is achievable with balloon kyphoplasty and short-term pedicle screw instrumentation. Preserving the epiphysis of the diseased vertebra and the immediately vertebral height restoration during the surgery retain the possibility for vertebral height recovery. Active surgical treatment is suggested for children who have vertebral plana brought on by spinal EG to maintain the ability to regain vertebral height.

## Data Availability

The raw data supporting the conclusions of this article will be made available by the authors, without undue reservation.
